# Making care more patient centered; experiences of healthcare professionals and patients with multimorbidity in the primary care setting

**DOI:** 10.1186/s12875-021-01420-0

**Published:** 2021-04-09

**Authors:** Sanne Jannick Kuipers, Anna Petra Nieboer, Jane Murray Cramm

**Affiliations:** Department of Socio-Medical Sciences, Erasmus School of Health Policy & Management, Erasmus University Rotterdam, Rotterdam, the Netherlands

**Keywords:** Patient-centered care, Multimorbidity, Primary care, General practice, Care delivery

## Abstract

**Background:**

The present study describes how primary care can be improved for patients with multimorbidity, based on the evaluation of a patient-centered care (PCC) improvement program designed to foster the eight PCC dimensions (patient preferences, information and education, access to care, physical comfort, coordination of care, continuity and transition, emotional support, and family and friends). This study characterizes the interventions implemented in practice as part of the PCC improvement program and describes the experiences of healthcare professionals and patients with the resulting PCC delivery.

**Methods:**

This study employed a mixed-methods design. Semi-structured interviews were conducted with nine general practitioners and nurse practitioners from seven primary care practices in Noord-Brabant, the Netherlands, that participated in the program (which included interventions and workshops). The qualitative interview data were examined using thematic analysis. A longitudinal survey was conducted with 138 patients with multimorbidity from these practices to assess perceived improvements in PCC and its underlying dimensions. Paired sample *t* tests were performed to compare survey responses obtained at a 1-year interval corresponding to program implementation.

**Results:**

The PCC improvement program is described, and themes necessary for PCC improvement according to healthcare professionals were generated [e.g. Aligning information to patients’ needs and backgrounds, adapting a coaching role]. PCC experiences of patients with multimorbidity improved significantly during the year in which the PCC interventions were implemented (*t* = 2.66, *p* = 0.005).

**Conclusion:**

This study revealed how primary PCC can be improved for patients with multimorbidity. It emphasizes the importance of investing in PCC improvement programs to tailor care delivery to heterogenous patients with multimorbidity with diverse care needs. This study generates new perspectives on care delivery and highlights opportunities for its improvement according to the eight dimensions of PCC for patients with multimorbidity in a primary care setting.

## Background

Primary care organizations throughout the world strive to make their care more patient centered, defined as “providing care that is respectful of and responsive to individual patient preferences, needs, and values and ensuring that patient values guide all clinical decisions” [1]. To achieve this goal, an organization must fulfill the eight dimensions of patient-centered care (PCC; also referred to as person-centered care) defined by the Picker Institute [2]: 1) patient preferences, 2) information and education, 3) access to care, 4) physical comfort, 5) coordination of care, 6) continuity and transition, 7) emotional support, and 8) family and friends (Table 1, Method section) [2–4]. Evidence for the effects of PCC provision is clear; healthcare organizations with higher dimensional PCC scores report better patient and organizational outcomes [5, 6]. However, despite international agreement about the importance of PCC, considerable consensus on its definition, and a common understanding of how it would ideally look, knowledge about the types of PCC interventions implemented in the primary care setting and about whether these interventions generate more positive patient experiences is insufficient [4].

Although primary PCC provision is desirable for all populations, it may be especially valuable for patients with multimorbidity. Multimorbidity is often described as the co-existence of two or more chronic conditions [7]. Patients with multimorbidity often report poor health and quality of life, functional impairment, and frailty, and have a greater risk of mortality [8–12]. Most primary care delivery follows single disease–oriented guidelines; multiple disease–oriented guidelines would be beneficial to avoid the fragmentation of care for patients with multimorbidity [13, 14]. Moreover, the complex care needs of these patients render their management very time consuming and expensive [15]. As care delivery must be tailored to their needs to improve outcomes, primary PCC is important for this patient population.

### Study objectives

In the present study, we aimed to describe how primary care could be improved for patients with multimorbidity by evaluating a program designed to improve PCC delivery to these patients in general practitioners’ (GPs’) practices in the Netherlands. The implementation of this program provided a unique opportunity to characterize PCC delivery in practice and healthcare professionals’ and patients’ experiences with this care. Specifically, the aims of this study are to 1) identify the interventions that were part of the PCC improvement program, 2) characterize the experiences of healthcare professionals with the program implementation, and 3) determine whether the program implementation is associated with more positive patient-centered experiences among patients with multimorbidity.

## Methods

### Setting

The “Zorggroep RCH Midden Brabant BV” is a cooperative that invests in the improvement of PCC delivery in the 160 primary care practices of its GP members in the Netherlands. In 2017, it started the PCC improvement program for patients with multimorbidity, based on the eight PCC dimensions (Table 1), in seven GP practices in Noord-Brabant that were considered to be most patient centered and known to be most enthusiastic about further improving PCC.

**Table 1:** The eight dimensions of patient-centered care

Patient preferences	Healthcare professionals treat patients with dignity and respect and involve them in decisions regarding their care. They support patients in setting and achieving treatment goals, e.g., via individualized care plans based on patients’ needs, wishes, and preferences
Information and education	To empower them to be in charge of their care, patients are informed about all aspects of their care and have access to their medical records. The information provided is suitable for all education levels, migration backgrounds, languages, and ages, among others. The need for informative and open communication between patients and healthcare professionals is recognized
Access to care	Healthcare is affordable, and medical buildings are easily accessible for all patients (including, e.g., those who are blind and those who use wheelchairs or walkers). Appointment scheduling is easy and wait times are short
Physical comfort	Healthcare professionals pay attention to patients’ physical comfort by, e.g., providing pain management and addressing sleep problems and shortness of breath. Physical comfort is optimized at the organizational level via the provision of comfortable, clean (waiting) rooms and sufficient privacy
Coordination of care	The organization’s team of healthcare professionals is well informed about the care delivered to their patients, and care delivery is well coordinated, e.g., via frequent team meetings. Patients know who is coordinating their care and whom they can contact when they have questions about their care
Continuity and transition	When multiple healthcare professionals are involved in care provision to a patient, they all transfer information regularly and adequately, and ensure that their care delivery and advice are well coordinated. When patients are referred to healthcare professionals in other disciplines, they know where to go and why
Emotional support	Healthcare professionals offer emotional support to patients when needed, by paying attention to patients’ possible fear, depression, and anxiety, and the impacts of chronic conditions on patients’ private lives. Patients are made aware of their ability to obtain emotional support, e.g., from social workers or peer groups
Family and friends	As many conditions impact not only patients, but also their family members and friends, healthcare professionals involve these individuals in the care process (with patients’ consent). They provide support and address any questions and needs regarding patients’ care

During one year of the PCC improvement program implementation in 2017 and 2018, healthcare professionals from the GP practices attended four meetings and several workshops covering a variety of patient-centered interventions (Table 2, Result section). The meetings focused primarily on increasing participants’ knowledge about the PCC dimensions, and provided opportunities for participants to reflect on and share their experiences with PCC implementation in practice. During the PCC improvement program, a toolbox of interventions was provided to the involved healthcare professionals, which were taught during workshops. Throughout the program, and in line with the concept of PCC, investment in a variety of interventions was emphasized, given the variation within individual GP practices and among patients with multimorbidity and their needs.

### Study design and data collection

A mixed-methods design was used to describe and evaluate the PCC improvement program and to capture the experiences of healthcare professionals and patients with multimorbidity. The qualitative data described the PCC improvement program and healthcare professionals’ experiences with it, and the quantitative data described improvements in patients’ experiences. The first author conducted interviews (~ one h each) with nine healthcare professionals who participated in the PCC improvement program (four GPs and five nurse practitioners, selected by purposive sampling). Ten interviews were scheduled, but one interview was cancelled due to the participant’s illness. The interviews were conducted at the GP practices in January and February 2018. All participants were familiar with the researcher, whom they had met at PCC improvement program meetings, and the goals of the study. The interviews were semi-structured according to the PCC dimensions. Only the researcher and interviewee were present during each session. The interviews were recorded digitally, with the participants’ permission, and transcribed verbatim. The researcher used a script to ensure consistency across interviews.

In order to identify whether the PCC improvement program was associated with more positive experiences among patients with multimorbidity, a survey was sent, at baseline (T0) and one  year later (T1), by mail to patients with multimorbidity [two or more registered conditions, i.e., asthma, chronic obstructive pulmonary disease (COPD), diabetes, heart and vascular conditions] from the participating practices. PCC experience was assessed using the 36-item patient-centered primary care instrument, validated for patients with multimorbidity [16]. Seven items of this instrument covered patient preferences; five items each covered physical comfort and access to care; four items each covered coordination of care, continuity and transition, emotional support, and information and education; and three items covered family and friends. Responses are given on a scale ranging from one (totally disagree) to five (totally agree), with higher mean scores indicating a greater degree of PCC. Average dimension scores were calculated in the presence of responses to at least two-thirds of items, and average total scores were calculated in the presence of at least six dimension scores. In this study, the Cronbach’s alpha value for this instrument at T0 and T1 was 0.96, indicating good reliability. We also asked participating patients to provide information on their background characteristics, such as age, gender, education level (1, primary education or less; 0, preparatory school for vocational secondary education or more), and marital status (1, living alone, widowed, or divorced; 0, married/living with partner).

### Data analysis

Inductive thematic analysis was performed with the interview data, as described by Braun and Clarke [17]. First, the interviews were transcribed verbatim and the full transcripts were read for familiarization with the data. Second, the transcript content was classified according to the eight dimensions of PCC. Using ATLAS.ti, (version 8.4.18; ATLAS.ti Scientific Software Development GmbH), the first author then coded and categorized the dimension-classified content. Finally, the authors generated themes that represented needs for PCC improvement in primary care for patients with multimorbidity in each dimension identified by healthcare professionals. All codes and themes were discussed among all of the authors until agreement was reached. The themes were also discussed during a meeting of all PCC improvement program participants. The healthcare professionals recognized all themes raised, and no additional theme emerged during this meeting.

For analysis of the patient survey data, descriptive statistics (means, ranges, standard deviations, frequencies, and percentages) were first generated for all variables. Only data of patients that filled in the questionnaire at both T0 and T1 were analyzed (*n* = 138). Then, we used paired-sample *t* tests to compare PCC total and dimension scores at T0 and T1. As improvement was expected, we conducted one-sided tests. The significance level was set at 0.05. Reliability was assessed using Cronbach’s alpha. The statistical analyses were performed using SPSS software (version 26; IBM Corporation, Armonk, NY, USA).

## Results

### Study participants

In total, 22 healthcare professionals from the seven GP practices participated in the PCC delivery improvement program. Nine of these professionals [four GPs and five nurse practitioners (NPs)] were interviewed. At T0, 413 patients were eligible to participate in the survey; 19 of these patients were excluded due to incorrect addresses (*n* = 5), death (*n* = 4), visual impairment (*n* = 3), recent moves with deregistration from the GP practices (*n* = 2), admission to a nursing home/hospice because of a terminal illness (*n* = 2), dementia/cognitive decline (*n* = 2), and hemorrhage (*n* = 1). Of the 394 remaining patients, 216 filled in the survey (55% response rate). Between T0 and T1, 59 patients dropped out because of death, admission to a nursing home, and deregistration from the GP practices. At T1, 335 patients were eligible to participate; 19 of these patients were excluded due to incorrect addresses (*n* = 5), death (*n* = 5), dementia/cognitive decline (*n* = 5), admission to a nursing home (*n* = 2), and inability to fill in the survey (*n* = 2). Of the remaining 315 patients, 169 filled in the survey (54% response rate). The overall attrition rate was 36%; 138 participants filled in the questionnaire at both T0 and T1.

### Intervention components of the PCC improvement program

During the PCC improvement program, a toolbox of interventions was provided to the involved healthcare professionals (Table 2). The healthcare professionals reported that participation in the PCC improvement program led them to select various interventions that they would like to implement. For example, health literacy recognition training was a priority intervention for a GP practice where many patients with immigrant backgrounds were treated, whereas other interventions were more important for other practices. The interventions of choice were explained and taught during multiple workshops. For example, an “evaluation of PCC on the job” workshop was held to help all participating healthcare professionals improve all eight dimensions of PCC; among other topics, practice interiors and privacy, documentation, management of wait times, and information provision during consultations were discussed. Other workshops aimed to contribute to the information and education dimension and facilitate informative, efficient and open communication between patients and healthcare professionals, such as by using the teach-back method, emphasizing the importance of listening to patients’ needs, checking whether they properly understand information, and adjusting information provision as needed, which is especially valuable for patients with low health literacy. All interventions offered during program implementation are described in Table 2.

**Table 2:** PCC interventions for healthcare professionals in the primary care setting

*Consultation videotaping*	A workshop aiming to improve the coaching role of healthcare professionals during consultations by discussing video recordings of consultations with patients
*Evaluation of PCC on the job*	A workshop aiming to help all healthcare professionals employed at an organization to improve their patient-centeredness. All daily care activities, from appointment making via internet/telephone to front desk work, provision of advice, and consultation structure, are evaluated
*Listening*	A workshop aiming to help healthcare professionals understand patients’ questions and needs at the start of consultation by listening to patients first, instead of immediately asking questions
*Motivational interviewing*	A training session in a directive, patient-centered approach to counseling that prepares patients for behavior changes. With motivational interviewing, attention is payed to building a strong patient–provider relationship and working toward patient autonomy and responsibility during the care process
*NIVEA*	A workshop aiming to help healthcare professionals avoid judgement or interpretation of patients’ feelings without asking for clarification or further information
*Shared decision making*	A workshop aiming to train healthcare professionals to use shared decision making during consultations to 1) prepare patients for the decision-making process (e.g., by informing them of consultation goals), 2) determine goals (e.g., jointly explore patients’ situations, share relevant medical information, and formulate goals), 3) agree on action points (e.g., by discussing all options), and 4) act and evaluate (e.g., by acting on agreements and reflecting on progression)
*Teach-back method*	A workshop in which healthcare professionals learn to always check whether patients fully understand the information provided by asking patients to explain/repeat what they have just been told. This approach provides healthcare professionals with better insight on whether their information provision is adjusted adequately to patients’ skills, and whether patients remember the right elements
*Three good questions*	An intervention based on a Dutch national campaign that aims to reassure patients that their wishes, anxieties, and needs matter during healthcare consultations. The ‘three good questions’ that patients can ask their healthcare professionals are 1) What are my options? 2) What are the pros and cons of those options? and 3) What does that mean in my situation?
*Topic list*	An intervention exploring areas in which patients need support. The topic list is sent to patients before consultations; it contains depictions of pain and topics such as stress and lack of sleep. The list makes patients aware of the range of topics that they can discuss with their healthcare professionals
*Training in illiteracy recognition*	A training session focusing on healthcare professionals’ recognition of illiterate patients and adjustment of their communication accordingly during consultations. The training also addresses such recognition by triage assistants and front desk staff when answering the telephone

*PCC* patient-centered care, *NIVEA* niet invullen voor een ander [do not interpret the feelings of a patient without asking]

### Experiences of healthcare professionals with PCC improvement

**Fig. 1:**
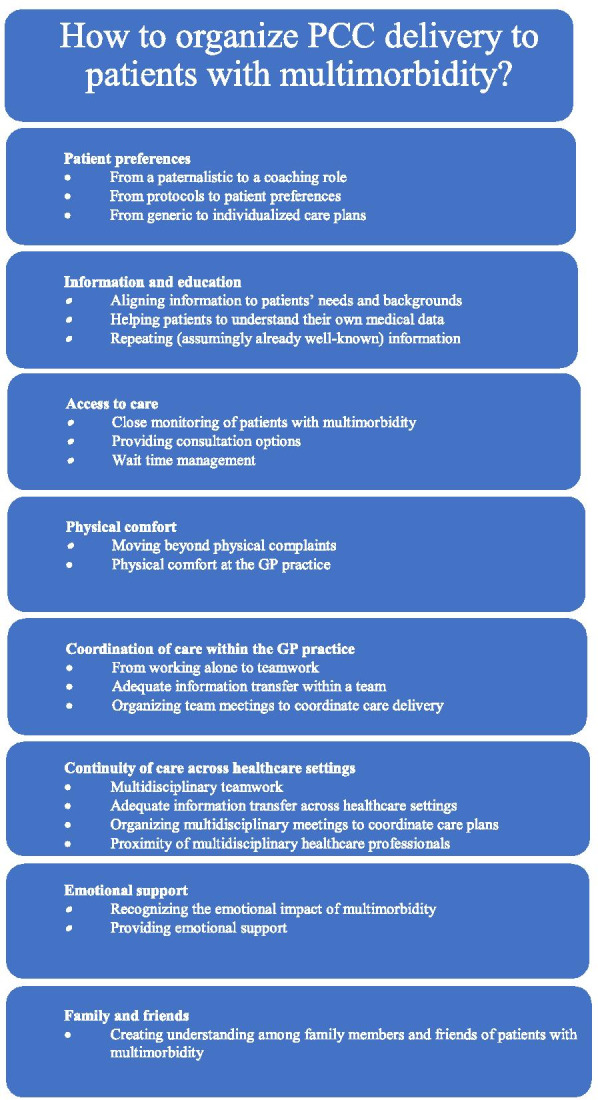
Overview of how to organize patient-centered care (PCC) for patients with multimorbidity

The healthcare professionals reported that the program meetings and interventions improved their PCC delivery. These improvements are described below according to the PCC dimensions, with the provision of supporting quotations from the NP and GP interviewees. The main themes are also depicted in Fig. 1.

#### Patient preferences

##### From a paternalistic to a coaching role

According to the healthcare professionals, PCC for patients with multimorbidity contributes to patients’ well-being when it is based on individuals’ wishes, needs, and abilities. Thus, the professionals felt that they should involve patients in their care and decision-making processes, and stimulate patients to set and achieve their own treatment goals*.* According to the GPs, this approach requires more of a coaching role than the paternalistic role that they used to play, with the aim of supporting individual patients’ achievement of their own goals.

“In the past, we used to let people come and draw blood for all kinds of tests, and thus, we thought, provided good care for that patient. And now we look more and more at what suits the patient; what does the patient need? Some measure several values themselves, such as blood pressure or sugar levels, and someone who is well regulated may not need to come as often as someone else. So it is more patient centered, meaning that the patients decide for themselves what their goals are and how often they need support, instead of us telling them ‘you have to come so often and this is what we are going to do.’ We have more of a coaching role now.” (GP7)

To be able to take on a coaching role, the healthcare professionals emphasized the importance of listening to patients first, as needs and wishes differ among patients. At the beginning of the program, the professionals believed that such listening was a basic communication skill that they already possessed, but during the program they found that it was more difficult than expected. The healthcare professionals learned to be silent at the start of consultations and to listen to patients’ needs for at least 1 min. They concluded that this approach is more efficient than one in which they begin by providing solutions without precisely understanding the problem.

“Suppose I sit here on the edge of my chair and do not let the patient finish, but immediately start asking counter-questions. The result is that he or she does not feel heard, because in the end I have not asked what he or she wants to know from me. Instead, I come up with solutions, without knowing what the real question is. The calmer you are, the more serenity you radiate, the more open you stay, the more information you get, and the faster it goes. That is the trick.” (GP1)

The healthcare professionals also reported that they send questionnaires covering disease-related topics to patients before follow-up consultations, to prompt them to think about what they wished to discuss with their GPs and/or NPs. The NPs reported that this approach helped patients formulate and express their individual preferences and needs, and made them more in charge of their care.

“I use this list especially with cardiovascular risk management and with diabetic patients. I usually tell them: ‘The list contains all kinds of aspects that can affect your health. Your illness, but also how you feel and how healthy you feel. Is there anything on this list that makes you think that is just something I would like to talk about, because I have a problem with that or I have a question about it?’" (NP2)

##### From protocols to patient preferences

According to the healthcare professionals, care delivery according to patients’ preferences requires flexibility concerning protocols and guidelines that they used to follow. For example, protocols mandate a fixed number of follow-up consultations per year for patients with diabetes, but some patients with multimorbidity prefer fewer follow-ups, as they consider themselves to be experts in diabetes, given that they have lived with it all of their lives and can manage everything themselves.

“With diabetes, we have check-ups four times a year, but a number of patients tell me: 'I have had diabetes for twenty years now. Everything has already been said, it is all going very well. I feel good, the check-ups are good, why do I have to come four times a year?" It used to be protocol based, but now we are reducing that number. ‘How many times would you like? When? Whom would you like to see?’” (GP1)

##### From generic to individualized care plans

The healthcare professionals reported that they had begun to formulate individualized care plans together with patients, stimulated by the goals of shared decision making and consideration of patients’ preferences, wishes, and needs. They reported that difficulties could arise when patients’ care preferences contradicted their own, but emphasized the importance of following the former, as long as all options and potential side effects are discussed.

“I will always explain why the protocol or standard says that a certain choice is best, but I do respect the patient's choice. As long as I have pointed out to the patient what the possible risks might be.” (NP3)

#### Information and education

##### Aligning information to patients’ needs and backgrounds

The healthcare professionals acknowledged that provision of the right information and education to patients is crucial. They recognized that patients’ levels of understanding/education and preferred form of information provision vary, rendering the alignment of information provision to individual patients important. They also acknowledged the difficulty of doing so, as patients’ health literacy and/or resources can be difficult to recognize. The program’s training in illiteracy recognition and the teach-back method helped them to recognize patients’ needs. These professionals also spoke of the importance of helping patients to distinguish trustworthy from unreliable sources, as patients also gather information elsewhere.

“I always ask the patients what they like; I can give information verbally or in a letter so that they can read it again later. I also ask them how they look for information themselves. One patient goes neurotically through all the forums, while another thinks you cannot look anything up on the internet. Then I tell them that it is good to use different sources of information, but that they have to assess the value of those sources. Some patients can do that and others cannot. So, I often help them to determine where to find relevant information.” (GP4)

##### Helping patients to understand their own medical data

The healthcare professionals realized that if patients had access to their own medical data, they had to help them understand it. Examples that facilitated patients’ understanding were the addition of “smileys” (color-coded happy/sad face icons) to medical records to indicate that values are (not) good, and the drawing of visual graphs during consultations.

“Patients do not know how to see if their values are good, but now there are smileys. An LDL of 2.5 comes with a green smiling face. And the patient is invited to email if it is orange or red. And if they want to email anyway, even when it is green, that is all right, if they still want confirmation. I think that is pretty much the future.” (NP3)

“Just the values, they do not understand of course. And they are not to blame, because those sugar values are developed internationally, and I find that difficult too. So, I try to show in graphs what happens, but not only with the sugar, also with the blood pressure or weight. And you can put two graphs together and then say ‘okay, your weight has risen, but your sugar rises along with it. When the weight goes down, the sugar goes down as well.’ So then one can see what is happening there. And that is a lot of fun actually.” (NP6)

##### Repeating (assumingly already well-known) information

The healthcare professionals emphasized the power of repetition for patients with multimorbidity. Although these patients’ conditions are chronic and they perform most actions (e.g., insulin injection) practically automatically, the professionals emphasized the value of checking whether the patients are still performing the actions correctly.

“I think that for some patients, things do become a habit, insulin therapy for example. Then it is wise to repeat it once more. Because you often assume that people know it all, that is also the case when using an inhaler with a spacer, for example. Now I often hand out again the leaflet that says how to do it. Then I will go through it briefly. And then they say ‘oh, I don’t actually do it like that,’ or they do not know that they have to clean the puffer as well. Even though they have done it like that for years, I just say it again.” (NP8)

#### Access to care

##### Close monitoring of patients with multimorbidity

The healthcare professionals emphasized the importance of closely monitoring all patients with multimorbidity to ensure that they receive the care they need. Thus, they reported that they schedule follow-up appointments (even those farther in the future, for patients who prefer less follow-up) directly after consultations. Reminders are sent, and patients who do not attend follow-up appointments are contacted by telephone to schedule new appointments.

“The people who I see always leave here with a new appointment. And if someone for example cancels an appointment through the assistant, they get a reminder; or if they don't show up, I call them. And if they do not answer, I send them a letter. At the time of their checkup every two months we monitor whether they are back in the picture again; if not, they will receive another reminder. And once every three months we also get an overview from the healthcare service provider, which also keeps track of when people are in danger of getting lost to follow-up.” (NP2)

##### Providing consultation options

The healthcare professionals reported that they offer a variety of (follow-up) consultation options to enable tailoring to patients’ preferences and needs, e.g., to account for their work hours or physical ability to come to the GP practice. Some consultations, e.g., those at which measurements must be taken, need to be conducted in person, but others, e.g., those held to provide information and regular check-ups, can be done by telephone or online.

“Telephone consultations are not just for my own convenience, but for both sides. If patients come here just to tell me something which takes two minutes, it is also annoying for the patient. And for me it just takes the same time when I can help them over the phone. I ask them sometimes: ‘you can come here, but we can also do it over the phone.’ We leave the choice up to the patient. Only a new blood pressure measurement, well, that is not possible over the phone.” (NP3)

“Some patients find it [consultation by email] very pleasurable. Other patients really just want the personal contact, either by phone or physically.” (GP5)

##### Wait time management

The healthcare professionals reported that their GP practices pay attention to patient wait times before in-person consultation and on the telephone. One practice improved this aspect by extending the hours during which they could be reached by telephone, and then closely monitoring telephone accessibility to determine where further improvement was needed. The professionals also emphasized the importance of expectation management by informing patients how long they must wait.

“To the annoyance of the assistants, our phone is always open. We could switch on the answering machine, but we do not want to. Even at lunchtime the phone is answered and we monitor it every day. Daily at five o’clock, I get a list with the day's waiting times. And once every week we have a meeting about the telephone times and we would like to see that more than 90% is answered within two minutes. The emergency line is always within 30 seconds.” (GP4)

“It helps to inform patients about the waiting times. When people see how long it takes, they know where they stand.” (NP9)

#### Physical comfort

##### Moving beyond physical complaints

The healthcare professionals reported that physical disease aspects, including comfort (e.g., pain, sleeping problems), were predominant topics of discussion, by the professionals and patients with multimorbidity, during consultations, as patients must cope with these aspects daily and as physical complaints are traditional foci of primary care delivery. With greater knowledge about PCC, however, the professionals realized that physical aspects differ among patients and are not the only components of physical comfort; they reported that they had begun to also ask, for example, about the suitability and comfort of use of the materials needed for disease management (e.g., insulin injection).

“I think that physical comfort has always been a goal in primary care. And as a nurse practitioner, you pay a lot of attention to what kind of obstacles patients experience as a result of their condition. And that is often somatically oriented, i.e., focused on physical comfort.” (NP2)

“Physical comfort often looms large in the patient's perception. That's what bothers them the most, so when they visit for check-ups, the first thing they say is 'I am in pain.' This is also much more important to them than all kinds of other factors that may be much worse compared to the pain. But they feel the pain now and that must be resolved now as well.” (GP4)

“There are a number of things that you ask by default, such as 'how short of breath have you been?,' 'does the cough bother you?,' 'does it interfere with your social contacts?.' But also, the questions to diabetics: 'Are your injections comfortable enough?,' 'Are your materials suitable?,' 'Do you sleep well?.' A lot is about physical comfort.” (NP2)

##### Physical comfort at the GP practice

During the study period, the healthcare professionals made many improvements to their practice interiors to ensure patients’ comfort and privacy, based on suggestions provided during the “evaluation of PCC on the job” workshop and those shared by other participants. Examples are the provision of comfortable chairs and creation of a nice atmosphere in the waiting room, and the separation of the front desk from the waiting room.

“We just have got everything brand new. We tested a lot of chairs, and we have got half of them with cushions and half without. All very washable, because people very quickly find it dirty. Everything is built according to the latest requirements; everything is easily accessible for wheelchairs, everything is height adjustable; for example, the examination table goes from very low to very high. All aisles are wide, and also in the corners there are special recesses for stretchers. The ambulance entrance is completely on a straight line that is the shortest possible route. The walking routes are such that people enter and leave as quietly as possible. And the partition at the reception desk is there so that others cannot see who they need to visit, be it a psychologist or a doctor. And next to the desk, there is also a special room, so that people cannot listen in, and if something private is asked at the desk. That is a soundproof room so they can get the results there. And of course, the large toilet facility for the disabled. And at the back of the toilet a hatch to deposit urine samples, so no one can see that you turn in pee.” (GP4)

“We have addressed the privacy issue, and in particular that you can overhear others. At first, the phone calls were audible in the waiting room, but after installing a glass wall this was a lot less.” (NP2)

“Learning about this aspect was really an experience. Sometimes you come across people who are very fat and are supposed to sit in such a small chair, but then say 'I will just stand here.' Now I realize it is not a comfortable chair for them.” (NP8)

#### Coordination of care within the GP practice

##### From working alone to teamwork

To improve the coordination of care within their GP practices (i.e., among GPs, NPs, front desk workers, and triage nurses involved in care delivery to patients with multimorbidity), and thus also improve patients’ satisfaction with care, the healthcare professionals recognized the importance of knowing what their colleagues are doing for patients; they also admitted that they did not always have this knowledge. Thus, they began to observe each other’s consultations to gain insight into colleagues’ expertise and contributions to patient care, which allowed them to better coordinate their own care delivery and/or ask for help.

“We would like to use each other's expertise. So, for example, an assistant walks into my office at lunchtime to ask me about a patient. Like, ‘would you like to help me determine how I could deal with this issue?’” (NP2)

##### Adequate transfer of information within a team

The healthcare professionals recognized the importance of all-encompassing documentation and efficient information transfer to achieve teamwork and adequate knowledge of all team members’ contributions. They noted that every aspect of patient care should be well documented in a system accessible to all healthcare professionals at the practice, who can read this information in preparing for patient consultations. For example, NPs explained that they prepared for consultations by reading GPs’ notes from previous consultations. Professionals from one GP practice mentioned that they also check each other’s work to avoid mishaps, not out of distrust, but mainly to make sure that documentation that is important for accurate care delivery is not overlooked.

“It is very important for me to see what the GP has written down. When someone comes for a consultation, I check in advance what has happened to that person since the last time I saw the patient. That may be on a completely different level, but all the information, including that from the consultations with the GP, is important.” (NP2)

“We also verify all phone calls with the assistants: everyone who called today is on the authorization list. Other practices do not do that, because they say 'but I trust my assistants.' I do trust my assistants, because we have really good assistants, but even still, things are not always as they should be.” (GP4)

##### Organizing team meetings to coordinate care delivery

The GPs and NPs reported that they often organized team meetings with all practice professionals to ensure that care is well coordinated. In one practice, daily morning meetings were held to align care delivery and discuss important content or questions that would likely arise during that day.

“The cooperation with the general practitioners is fine. We casually enter each other's rooms or I schedule a brief telephone consultation, and there is a structured low-threshold team meeting with the general practitioners and also with the assistants. They do not have anything to do with chronic care, but they do plan the appointments and receive patients.” (NP9)

“I always prepare my agenda; I check all the results in advance or any items I already promised I would discuss. Every morning we go through all that. Every day we have a start-of-day meeting with the whole team. And also with the assistants present. Details about patients or the practice are discussed there.” (NP3)

#### Continuity and transition among healthcare disciplines

##### Multidisciplinary teamwork

The professionals recognized that in the provision of care to patients with multimorbidity, working with care providers from disciplines (e.g., psychologists, dieticians, physiotherapists, hospital specialists, social workers) strengthens the continuity of care and aids the detection of patients’ problems and the delivery of tailored care.

“It is nice that when you are worried about something you can ask 'gosh think along, do you have any other points of view?’ That you just start thinking along from your own expertise. Because a psychologist might think very differently from a psychiatrist and a nurse practitioner.” (GP7)

“In cooperation with the district nursing service, we get a much better understanding of what the most profound problems are for a patient in a home situation. Sometimes this is not necessarily pain or shortness of breath, but for example, no good contact with the children anymore or loneliness or no daytime activities at all, or that the house is neglected. And that way you can take a much broader look at what that patient needs.” (GP5)

##### Adequate information transfer across healthcare settings

Similar to the need for an efficient intra-practice information system, the healthcare professionals indicated the importance of efficient information transfer among all healthcare organizations involved in the care of patients with multimorbidity. They reported that they used a chain-like system in which all involved professionals shared information about their part of care delivery to individual patients, which helped to coordinate care and ensure its continuity.

“The other professionals do not literally see the information in our system. However, they can communicate through our chain information system. This is an automation system for communicating with chain partners. And you can open up bits of information and close up bits of information, so that only relevant information goes to the healthcare professional who needs it.” (GP1)

##### Organizing multidisciplinary meetings to coordinate care plans

To stimulate care continuity, the healthcare professionals organized meetings with professionals from other involved healthcare disciplines. During these meetings, they used each other’s expertise to coordinate care plans and discuss patients’ progress.

“We start by making a care plan and then the various disciplines are complementing. Various people may well contribute something. I myself, a doctor, a geriatrics specialist, and also home care can contribute something. It is solution focused, but also thinking along. That's how we try to complete the picture.” (NP2)

##### Proximity of multidisciplinary healthcare professionals

The healthcare professionals indicated that multidisciplinary teamwork is more efficient when other involved professionals work in the same neighborhood, village, or metropolitan area.

“We are really trying to work together with professionals in the neighborhood. So, we do not go to someone on the other side of town, because that does not work.” (NP8)

“I must say that the lines with the district nurses and the paramedics are actually very short, because here in a village you actually know everyone. We meet once a month in a home team meeting where we also specifically highlight the vulnerable patients.” (GP5)

#### Emotional support

##### Recognizing the emotional impact of multimorbidity

Although the healthcare professionals indicated that emotional support was not a regular topic of discussion during consultations, and that such discussions occurred more often with NPs than with GPs, they emphasized the importance of considering the potential impacts of chronic conditions on the feelings and private lives of patients with multimorbidity, as these effects may influence clinical aspects of patients’ conditions. They acknowledged, however, that they sometimes struggled with discussing emotional aspects. They indicated that they used the consultation time primarily to discuss all physical aspects accompanying chronic conditions, especially for patients with multimorbidity. The professionals reported that the use of topic lists provided more insight into the emotional aspects important to patients.

“It is a well-known fact that if people with diabetes are very emotional or stressed, then those sugar values can go up.” (NP8).

“Someone with severe COPD and rheumatism can be very limited in his mobility. Such a patient can also become very sad. Therefore, I think that a lot of people also find a sense of support and comfort important. Sometimes patients really develop depression. Of course, you must talk about that too.” (GP5)

“Yes, any chronic care protocol includes a section on how the patient experiences his illness, how he deals with it. So, it is part of it. It's just a tricky part. Because sometimes it can take a lot of time to go into it deeply.” (GP1)

##### Providing emotional support

The healthcare professionals noted that the emotional support that patients are determined to need is often provided by mental health NPs. They also emphasized the importance of adjusting this support to accommodate patients’ needs and preferences, as emotional problems can be difficult to discuss; trust is very important. The healthcare professionals reported that they sometimes struggled with the provision of adequate emotional support, and thus actively sought other resources (e.g., peer support groups, social workers, and psychologists) or helped patients to do so based on their wishes and needs.

“We have different mental health nurse practitioners, with different backgrounds. On purpose actually. So that for certain cases we have the option to better assess who suits whom. We have both a man and a woman. We have a psychologist, a social worker, and we have a psychiatric nurse.” (GP7)

“There are a number of people with COPD here who work out with the physiotherapists twice a week in a group, and get a lot of emotional support from it. From the other people in the group, but also from the physiotherapist. It just shows that everyone finds support in something or with someone else. As long as there is enough variety, so that people eventually end up somewhere where they feel supported.” (GP5)

#### Family and friends

##### Creating understanding among family members and friends of patients with multimorbidity

The healthcare professionals agreed that the involvement of family members and friends in the delivery of care to patients with multimorbidity is important, mainly because chronic illnesses are part of these patients’ lives. They also noted that helping people close to patients understand the patients’ conditions is important. However, the healthcare professionals acknowledged that they did not always try to achieve such involvement, as they struggled with the determination of when patients would like their family members or friends to be involved, and whether relatives have questions or needs concerning care delivery. The healthcare professionals reported various ways in which they involved relatives in care delivery, such as by making house visits to map out patients’ situations and asking whether relatives can attend consultations; in addition, they noted that relatives sometimes took the initiative in contacting the healthcare professionals.

“We know that patients spend less than 1% of their time here in the consulting room and spend much more time at home with family and friends.” (GP5)

“Family needs to come along to the consultation as well and be educated in order to understand why something is important. Someone with heart failure, for example, should not eat salt. But then the food does not taste good. So, then I explain to the family member who is cooking what it means if he/she always adds salt to the food, and that this can lead to a hospital visit. It is also a matter of great ignorance in the family.” (GP4)

Overall, the healthcare professionals felt that the PCC improvement program, including the intervention toolbox and workshops, generated new perspectives on care delivery and options for the improvement of the eight dimensions of PCC. In the next paragraph, we discuss whether these improvements yielded more positive PCC experiences for patients with multimorbidity.

### Experiences of patients with multimorbidity with PCC improvement

The mean age of the 138 patients who filled in the questionnaire at T0 and T1 was 73.50 (range, 48.45–94.32) years; 42.2% were male, 37.2% were single, and 33.8% had low educational levels. Table 3 shows that the patients perceived that PCC improved significantly over the study period (*t*(109) = 2.66, *p* = 0.005). Specifically, they perceived significant improvement in the physical comfort (*t*(117) = 1.80, *p* = 0.037), emotional support (*t*(122) = 2.35, *p* = 0.010), continuity and transition (*t*(86) = 2.37, *p* = 0.010), and family and friends (*t*(41) = 2.20, *p* = 0.017) dimensions (Table 3). Improvement of the coordination of care dimension was only marginally significant (*t*(115) = 1.51, *p* = 0.068). Patient preferences, access to care and information and education did not improve over time (*t*(133) = 0.44, *p* = 0.332; *t*(129) =—0.54, *p* = 0.296; *t*(132) = 0.54, *p* = 0.294, respectively).

**Table 3:** Patient-perceived quality of primary patient-centered care

Dimension	*n*	Score (mean ± standard deviation)		T0 vs. T1		
		**T0**	**T1**	***t***	**df**	***p***
Overall	110	3.90 ± 0.49	4.03 ± 0.43	2.66	109	0.005
Patient preferences	134	4.05 ± 0.61	4.07 ± 0.56	0.44	133	0.332
Physical comfort	118	3.96 ± 0.59	4.07 ± 0.49	1.80	117	0.037
Coordination of care	116	3.97 ± 0.61	4.06 ± 0.52	1.51	115	0.068
Emotional support	123	3.55 ± 0.74	3.73 ± 0.69	2.35	122	0.010
Access to care	130	4.12 ± 0.57	4.10 ± 0.49	-0.54	129	0.296
Continuity and transition	87	4.05 ± 0.59	4.21 ± 0.51	2.37	86	0.010
Information and education	133	3.97 ± 0.56	4.00 ± 0.46	0.54	132	0.294
Family and friends	42	3.72 ± 1.07	4.08 ± 0.76	2.20	41	0.017

## Discussion

With the present study, we aimed to describe how primary PCC for patients with multimorbidity can be improved by evaluating a PCC improvement program implemented in the Netherlands using a mixed-methods design; the qualitative data describe how PCC can be improved according to healthcare professionals, and the quantitative data describe whether patients with multimorbidity experienced improvements during the implementation of the PCC improvement program.

Our findings emphasize the importance of investing in PCC improvement programs, including the provision of an intervention “toolbox” and workshops, for the tailoring of care delivery to a heterogenous population of patients with multimorbidity with diverse care needs. They are in line with findings suggesting that the PCC dimensions are not equally important to all patients with multimorbidity, and that subgroups of these patients can be identified based on care needs [18, 19]. We found that the program provided healthcare professionals with new perspectives on care delivery and opportunities to make improvements in the eight PCC dimensions. These professionals’ experiences with PCC improvement were not correlated one-to-one with the interventions implemented but were closely tied to all lessons learned during program participation. The changes implemented in GP practices based on the program also improved patients with multimorbidity’s experiences with PCC delivery. As patient experiences have been associated with clinical effectiveness, patient safety, and health outcomes this is relevant for further improving care delivery for patients with multimorbidity [20].

This study shows that within the patient preferences dimension of PCC it is important that healthcare professionals adopt a coaching role to support patients’ goal achievement, listen to patient preferences, and formulate individualized care plans. The adoption of these practices does not mean that PCC cannot be evidence based; the two approaches can be integrated, although the manner in which this is done may differ among organizations [21]. For example, healthcare professionals should continue to discuss disease guidelines based on strong evidence for specific treatment options with patients.

According to this study, to improve the information and education dimension, the alignment of information to patients’ needs and backgrounds, helping patients to understand their medical data, and repeating (assumed to be well-known) information is important. Although the healthcare professionals emphasized the importance of supporting patients in being in charge of their own care, many patients with multimorbidity have reported that they feel unable to oversee all aspects of their care and that they require support [22]. However, our patient survey revealed that patients participating in this study did not experience significant improvement in this dimension, thus further improvement is needed.

Regarding the access to care dimension, healthcare professionals in this study recognized the importance of close patient monitoring, the provision of various consultation options, and the management of wait times. These findings are in line with the reported preferences of patients with multimorbidity, who have been found to make appointments with their GPs only when their symptoms are beyond their self-management abilities; thus, they prefer quick access to their care providers, and for some preferably via email instead of telephone due to long telephone wait times [23].

Identified themes in the physical comfort dimension include healthcare professionals’ recognition that they devote the majority of their attention to the clinical aspects of disease during consultations, as chronic conditions are often accompanied by pain, shortness of breath, and lack of sleep [24, 25]. However, according to this study, providing physical comfort also entails suitability and comfort of the materials needed for disease management. The healthcare professionals also reported that they had made many improvements to their practice facilities related to patients’ physical comfort and privacy, based on the PCC improvement program content. Previous studies have revealed the importance of the waiting room physical environment in primary care for the quality of care and patients’ satisfaction with care [26]. Indeed, in this study, patients’ survey responses indicated that the GP practice improvements improved their experiences.

Themes related to the coordination of care within the GP practice raised by the healthcare professionals include the need for teamwork and efficient information transfer, including the holding of practice-wide meetings. These findings are in line with relational coordination theory, which holds that effective coordination depends on the mutually reinforcing interaction of (timely, frequently, accurate, and problem-solving) communication and relationships (based on shared goals, shared knowledge, and mutual respect) between service providers [27]. For example, healthcare professionals’ knowledge of each other’s contributions to care delivery leads to mutual respect, frequent team meetings entail frequent and timely communication leading to shared goals, and efficient information transfer provides shared knowledge and accurate communication. The patients with multimorbidity surveyed in this study, however, perceived only marginal improvement in the coordination of care. Future research should focus on how patient experiences with coordination of care can be improved further.

Similarly, healthcare professionals participating in this study reported their efforts to work in multidisciplinary teams, with frequent meetings of all professionals involved in individual patients’ care to coordinate care plans, and multi-disciplinary information transfer; these aspects fall within the continuity and transition dimension of PCC*.* The continuity of care is often found to improve patients’ outcomes and satisfaction with care [28], and is typically managed mainly by GPs as the clinical leaders of multidisciplinary teams [29]. Frequent discussion of this role and its importance during the PCC improvement program may have led to GPs’ improved adherence to the role, and thereby the improved organization of multidisciplinary teamwork. This inference is supported by patients’ indication of significant improvement in the continuity and transition dimension of PCC in their survey responses.

The healthcare professionals stressed the importance of emotional support, a PCC dimension, as patients’ multiple chronic conditions often affect their private lives and social relationships; at the same time, they acknowledged that they had difficulty discussing such topics and providing adequate support. Research has shown that multimorbidity is often accompanied by anxiety and depression [30, 31]. The professionals’ difficulty with this dimension may reflect their lack of initial training in asking patients about their general emotional status, and/or due to patients’ reluctance to discuss their emotional problems. The latter is in line with the findings that stigma often prevents patients from disclosing emotional problems to their healthcare professionals, and that patients do not always believe that their GPs can adequately manage these problems [32, 33]. Although patients’ responses to the survey conducted as part of this study show significant improvement in this dimension, the emotional support score was lower than scores for other dimensions at T1, indicating that further improvement is needed. These results suggest that the emotional support component of the PCC improvement program examined in this study was insufficient. Several potential interventions targeting emotional support have been described: they include multiple peer support interventions for patients with chronic conditions [34] and effective self-management support interventions that include strategies for coping with stress and chronic conditions [35]. Future research should investigate whether the implementation of similar interventions would result in (further) PCC improvement, as perceived by patients with multimorbidity, and whether patients expect GPs and NPs to treat emotional aspects of their status, or whether taking these problems seriously and providing options for treatment elsewhere would be sufficient.

Finally, regarding the family and friends’ dimension of PCC, improving PCC includes the importance of helping patients’ family members and friends understand the patients’ conditions and potentially play roles in care delivery. Although the PCC improvement program did not include a workshop on the involvement of relatives in care delivery, significantly improved patient experiences were found in this dimension, presumably due to healthcare professionals’ improved application in practice after acquiring knowledge and theory-based perspectives. Interventions entailing strategies to involve family members and friends, among others described in a systematic review of family-centered approaches for adults with chronic conditions [36], may further improve the experiences of patients with multimorbidity.

In addition to identifying components needed for the improvement of the eight dimensions of PCC, the healthcare professionals who participated in this study identified difficulties with PCC delivery, such as the adjustment of information provision and education to patients’ needs and the provision of adequate emotional support. Although this study was not designed to explicitly describe barriers to PCC delivery, those described in the literature include healthcare professionals’ lack of knowledge, skills, and time [37]. Future research should investigate whether these barriers also apply in the implementation of the PCC improvement program examined in this study.

In summary, this study yielded a characterization of how primary PCC can be improved. The overview of the interventions implemented could be useful for GP practices aiming to invest in PCC. Furthermore, healthcare professionals’ descriptions of their experiences provided insight into the nature of PCC for patients with multimorbidity in practice. Survey data showed that the PCC experiences of patients with multimorbidity improved significantly during the year in which the PCC interventions were implemented, demonstrating the value of the program as a guiding framework for the further improvement of PCC delivery to these patients.

### Study limitations and suggestions for future research

Several limitations of this study should be considered when interpreting its results. First, as the program examined was implemented in the Noord-Brabant region of the Netherlands, the generalizability of the results may be limited. Future research should investigate the experiences of healthcare professionals and patients with the implementation of similar PCC improvement programs in other regions and countries. Second, although patients perceived a significant overall improvement in PCC, their survey responses showed no significant improvement in the information and education, access to care, or patient preferences dimension. These results may be explained by the ceiling effect, as the GP practices that participated in this program are among the best-performing practices in their region, with high baseline PCC scores and little room for improvement on a 1–5 Likert scale. The program may yield even better results in GP practices with lower baseline PCC scores; future research should investigate its implementation in average- and low-scoring (Dutch) GP practices. Third, the study design did not allow for the testing of direct relationships between interventions and outcomes. Given the goal of the PCC improvement program, this was not a study aim; however, the program’s intervention “toolbox” is not exhaustive, and interventions can be added and/or removed according to specific GP practices’ needs. Fourth, confounding variables may have influenced patients’ experiences with PCC during the 1-year study period. However, taking into account the efforts made by the healthcare professionals, investments made in the improvement of PCC were likely the main contributors to the observed improvements in patient experiences. Finally, the Netherlands has a strong primary care system [38] which is a prerequisite for PCC development [39]. This should be taken into account while determining the applicability of the PCC improvement program in other countries.

## Conclusion

The findings of this study indicate how primary care can be improved for patients with multimorbidity in the Netherlands, including interventions and a focus on PCC themes identified by healthcare professionals. PCC experiences of patients with multimorbidity improved significantly during the year in which the PCC interventions were implemented. The results of this study are valuable for the further improvement of PCC delivery to patients with multimorbidity in the primary care setting.

## Data Availability

The data are available upon (reasonable) request.

## Abbreviations

COPD: Chronic obstructive pulmonary disease
GP: General practitioner
NP: Nurse practitioner
PCC: Patient-centered care
